# The Superfund Basic Research Program—Research for the Future

**Published:** 2006-08

It is a time of introspection for the Superfund Basic Research Program, a university-based grants program established in 1987. While maintaining the program’s premise of supporting basic research for practical application to address the nation’s problems associated with hazardous waste sites, the program is continuously evolving and developing new approaches to address these problems. A course was set to allow the Superfund Basic Research Program to award grants annually and to diversify funding mechanisms. With this announcement of the results of the most recent competition, the program is now poised to meet these goals.

We are pleased to announce that NIEHS has made awards to six programs addressing complex issues associated with hazardous waste sites through interdisciplinary, multi-project team approaches. Included in these six awards are one new grantee, the University of Iowa, and five grantees that have been part of the program in the past: Columbia University, Michigan State University, the University of California at Berkeley, the University of North Carolina at Chapel Hill, and the University of Washington.

In their new research endeavors, these grantees are using integrated research models to understand how contaminants are transformed as they move through soils, sediments, and groundwater and how they interact with ecosystems and ultimately effect human health. These robust research efforts are augmented with graduate and postdoctoral training in interdisciplinary environmental research and with community outreach activities. Each grantee is also required to engage proactively in research translation to other federal agencies, industry, and the community at large.

In addition to these six awards, the program is also pleased to announce that it is initiating an Individual Research Project Program, which will support single investigator–initiated research. This initiative will allow the program to respond quickly to emerging issues and to fulfill identified unmet needs.

For interested investigators who are not already part of this vital and exciting program but would like to be, please contact program staff about future opportunities. It is not too early to start the planning process! The next solicitation is scheduled for release in August; subsequent solicitations will be announced annually in August.

We are available to assist you as you begin to conceptualize a future program at your university!

For more information on the Superfund Basic Research Program see: **http://www-apps.niehs.nih.gov/sbrp/**

## Contacts

**William Suk, Ph.D., Director.** | suk@niehs.nih.gov

**Claudia Thompson, Ph.D.** | thomps14@niehs.nih.gov

**Beth Anderson** | tainer@niehs.nih.gov

## Figures and Tables

**Figure:**
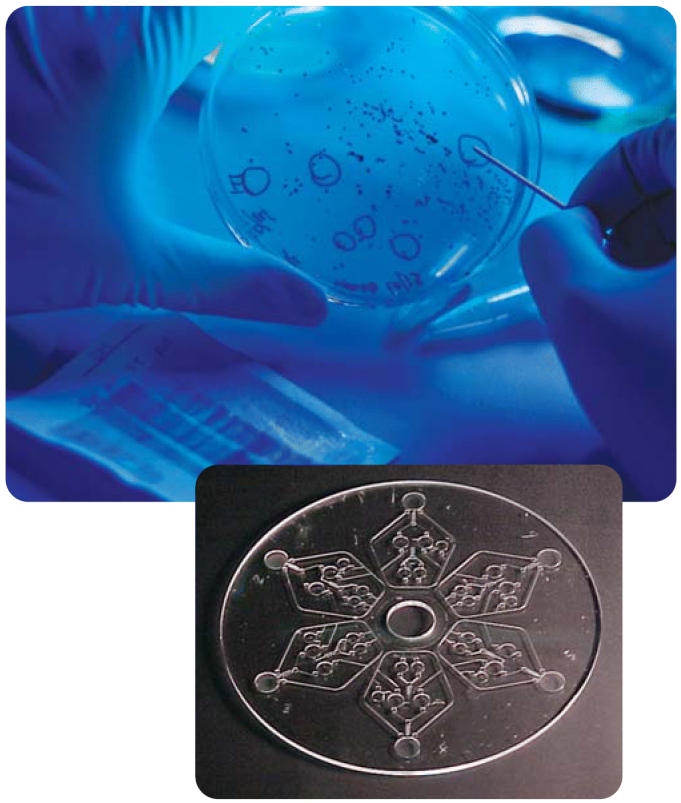
Microfluidic platform in a CD format for PCB analysis

